# Risk Scoring System to Predict Mortality in Gastric Cancer with Peritoneal Carcinomatosis

**DOI:** 10.3390/medsci12020030

**Published:** 2024-06-09

**Authors:** Marina Alessandra Pereira, Marcus Fernando Kodama Pertille Ramos, Amir Zeide Charruf, André Roncon Dias, Ulysses Ribeiro

**Affiliations:** 1 Department of Gastroenterology, Instituto do Cancer, Hospital das Clínicas da Faculdade de Medicina da Universidade de São Paulo, São Paulo 01249-000, Brazil; marcus.kodama@hc.fm.usp.br (M.F.K.P.R.); amir@colosaude.com.br (A.Z.C.); andre.dias@hc.fm.usp.br (A.R.D.);; 2 Department of Gastroenterology, Instituto do Cancer, Hospital das Clinicas HCFMUSP, Faculdade de Medicina, Universidade de Sao Paulo, Av. Dr. Arnaldo, 251, São Paulo 01246-000, Brazil

**Keywords:** stomach neoplasms, gastric cancer, peritoneal carcinomatosis, metastasis, survival, peritoneal metastasis

## Abstract

Gastric cancer (GC) with peritoneal carcinomatosis (PC) has a particularly unfavorable prognosis. This limited survival raises doubts about which factors confer an extremely worse outcome and which patients could benefit from more aggressive treatments, in an attempt to improve survival and better control the disease. This study aimed to evaluate the survival outcomes of patients with PC due to GC and develop a prognostic score to predict 6-month mortality. We performed an analysis of clinical stage IV GC with PC. Scores were assigned to risk factors and calculated for each patient from nine variables. Among 326 IVB GC, 211 (64.7%) had PC and were included. After calculating the score, 136 (64.5%) GCs were classified as a low-risk group and 75 (35.5%) as a high-risk group. Median OS was 7.9 and 1.9 months for low- and high-risk patients (*p* < 0.001). In the high-risk group, 77.3% of the patients died in <6 mo (*p* < 0.001). Palliative surgery and chemotherapy were associated with better survival, and the prognostic groups maintained statistical significance even when the same type of treatment was performed. In conclusion, the scoring system developed with variables related to patient performance status and clinical data was able to distinguish GC with PC with a high risk of 6-month mortality. Accordingly, verifying and validating our findings in a large cohort of patients is necessary to confirm and guarantee the external validation of the results.

## 1. Introduction

Gastric cancer (GC) is one of the most common cancers worldwide and represents the third most common cause of cancer-related deaths [1] For locally advanced GC, gastrectomy with D2 lymphadenectomy is the standard treatment with curative intent [2] Unfortunately, the majority of patients are diagnosed at an advanced stage, with signs of systemic disease [2,3].

The peritoneal cavity is one of the main sites of metastasis in GC and, at the time of diagnosis, 15 to 30% of patients present peritoneal metastases (PMs) [4,5]. The presence of peritoneal carcinomatosis (PC) generally indicates a particularly unfavorable prognosis, with no alternative curative treatment and scarce therapeutic options. Palliative systemic chemotherapy (CMT) represents the current standard of care for metastatic GC [2,6]. However, although the prognosis of stage IV GC has improved as a result of new chemotherapeutic and molecular targeting agents [7,8], the outcomes for patients who develop peritoneal carcinomatosis remain unsatisfactory, with an estimated median overall survival (OS) of 6 months [9,10].

Recently, in light of the need for additional therapies in the setting of peritoneal dissemination, the evolution and refinement of treatment options—such as cytoreductive surgery (CRS), hyperthermic intraperitoneal chemotherapy (HIPEC), pressurized intraperitoneal aerosol chemotherapy (PIPAC), and normothermic intraperitoneal chemotherapy—has generated new interest in the treatment of gastric PM [4,9,11,12]. Conversion therapy emerged as an alternative treatment for these patients, which included the administration of systemic CMT with intraperitoneal CMT followed by surgery [13,14]. This option has achieved promising results for these patients, with complete resection of the tumor and associated lesions (R0) [15,16,17]. However, the criteria to identify who among these patients may benefit from a treatment approach that may confer better survival remains unknown [18,19].

Therefore, improving the knowledge about PM—including prognostic factors and oncological outcomes—may contribute to a tailored approach in the treatment of patients with GC since the limited survival raises doubts about which factors confer an extremely worse outcome. Thus, the aim of this study was to evaluate the characteristics and survival outcomes of stage IV GC with PC and develop a prognostic score based on clinical and tumor variables for 6-month mortality to stratify these patients.

## 2. Materials and Methods

All patients with GC who underwent surgical procedures at our institution between 2009 and 2022 were retrospectively evaluated from a prospectively maintained database. Patients with a primary gastric tumor, histological diagnosis of adenocarcinoma, and stage IV GC with PC were considered eligible. Emergency surgery, patients with a history of appendix or ovarian neoplasms, recurrent tumors, patients who received intraperitoneal CMT or immunotherapy, and patients alive with less than 6 months of follow-up were excluded.

The clinical and pathological data collected included age, sex, body mass index (BMI), hemoglobin and serum albumin, neutrophil–lymphocyte ratio (NLR), American Society of Anesthesiologists (ASA) classification, Charlson–Deyo Comorbidity Index (CCI) [20] (without including age and GC as comorbidity), Lauren’s histological type, degree of tumor differentiation, and size and location of the tumor.

Abdominal and pelvis computed tomography (CT), endoscopy with biopsy, and laboratory tests were assessed preoperatively in all patients. The presence of peritoneal metastasis in patients was confirmed through diagnostic laparoscopy with biopsy of peritoneal lesions, including intraoperative frozen section examination, and/or peritoneal washing cytology. The staging was based on the eighth edition of the TNM/UICC [21].

The treatment adopted for each case was decided by a multidisciplinary team composed of surgeons, oncologists, radiologists, and pathologists. The surgical treatment adopted involved palliative gastrectomy, cytoreduction, gastric bypass (gastrojejunostomy/gastric partition), jejunostomy, or diagnostic laparoscopy alone. Gastrectomy and lymph node dissection (D1 or D2) were performed according to the guidelines of the Japanese Gastric Cancer Association [2]. Postoperative complications (POCs) were graded according to the Clavien–Dindo classification, and Clavien > 2 was defined as a major POC. All patients were operated on in a high-volume center by specialized surgeons.

Palliative chemotherapy (CMT) consisted of a doublet containing fluoropyrimidine (capecitabine or 5-fluorouracil) and a platin (oxaliplatin or cisplatin) as the preferred systemic regimen for the first line. In some cases, irinotecan and cisplatin CMT were chosen to avoid the use of infusion pumps or for those patients with difficulty swallowing capecitabine pills. For the second line, paclitaxel or irinotecan was used.

Follow-up was performed every month or in a shorter period, if necessary. This study was approved by our hospital’s ethics committee (CAAE: 26306419.8.0000.0065).

### Statistical Analysis

Descriptive statistics included frequencies with percent for nominal variables and mean (with standard deviation, SD) or median (with interquartile range, IQR) for continuous variables. A comparison of clinicopathological characteristics was performed using the chi-square test for categorical variables and the *t*-test for continuous variables.

The score was built with death within 6 months as the main outcome. The variables to compose the score were selected based on their statistical significance and clinical impact already known in the literature [3,22]. A multivariable binary logistic regression was used to assess the characteristics associated with 6-month mortality. Scores were assigned to each risk factor based on beta coefficients and calculated for each patient from all variables selected to define the score categories.

The receiver operating characteristic (ROC) with the area under the curve (AUC) was used to evaluate the performance metric of the score in predicting 6-month mortality. The optimal cutoff value was determined by maximizing Youden’s index (sensitivity + specificity—1). Patients were divided into “low-risk” and “high-risk” groups for 6-month mortality based on the cutoff value.

Overall survival (OS) was estimated using the Kaplan–Meier method, and the comparison of curves was completed using the log-rank test. Survival time was calculated from the date of diagnosis until the date of death or the last contact. A *p*-value of <0.05 was considered statistically significant. Statistical analyses were performed using SPSS software, version 20.0 (SPSS Inc., Chicago, IL, USA).

## 3. Results

Among 326 IVB GCs diagnosed in the referenced period, 211 (64.7%) had PC and were enrolled in this study. The mean age was 60.5 years old (±12.8, range 24–83), and the majority of patients were male (61.1%).

According to the surgical procedure performed, 53 (25.1%) patients underwent gastric bypass (34 gastrojejunostomy and 19 gastric partition); 55 (26.1%) underwent jejunostomy; 28 (14.7%) underwent gastrectomy (15 total and 13 subtotal), 6 underwent cytoreduction (2.8%), and the remaining 69 (32.7%) underwent only diagnostic laparoscopy. According to the site of metastasis, 86.3% had PM exclusively, and the remaining 13.7% also had metastasis in another site besides the peritoneum as follows: 19 hepatic, three distant lymph node, one pulmonary, and six ovarian metastases (corresponding to the cases that underwent cytoreduction). The clinical and surgical characteristics of all patients are presented in Supplementary Table S1.

The median length of hospital stay was 4 days (IQR 2–8), and major postoperative complications (POCs) occurred in 25 (11.8%) of cases. Excluding cases that only underwent diagnostic laparoscopy, the rate of POCs was 17.6%. A total of 64.9% of the patients were referred to palliative CMT, having received at least the first line of treatment. Furthermore, considering patients according to the occurrence of POCs, 70.4% (131/186) and 24% (6/19) of patients without POCs and with major POCs received palliative CMT (*p* < 0.001).

Of the 211 patients, 115 (54.5%) died in less than 6 months. Of the remaining 96 (45.5%) patients alive, 82 died after 6 months, and 14 remained alive at the time of this study.

### 3.1. Risk Scoring System for 6-Month Mortality

After analysis of risk factors related to death within 6 months (Supplementary Table S2), the following points were assigned to the variables to build the score: age ≥ 60 y (2 pts); female (2 pts); ASA III/IV (1 pt); CCI ≥ 1 (1 pt); hemoglobin < 11 g/dL (2 pts); NLR ≥ 3.85 (3 pts); ascites on CT scan (3 pts); stenosis on CT scan (2 pts); and tumor size ≥8 cm (1 pt) (Supplementary Table S3).

**Figure 1:**
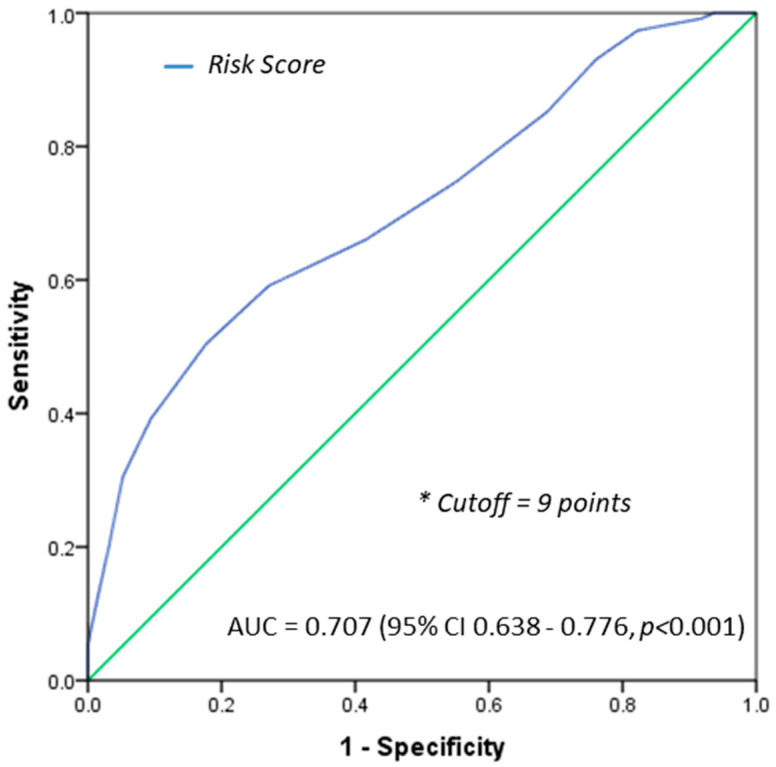
Receiver operating characteristic (ROC) curves for the 6-month mortality score. The area under the receiver operating characteristic curve (AUC) was 0.71, with an optimal cutoff value of 9 points.

The performance metric for risk score (maximum of 17 pts) derived from these pooled parameters was assessed through the construction of the ROC curve (Figure 1). The AUC for the 6-month mortality score was 0.707 (95% CI 0.638–0.776, *p* < 0.001), and the optimal cutoff value was 9 points (pts). Accordingly, 136 (64.5%) GCs were classified in the low-risk group and 75 (35.5%) in the high-risk group.

All nine variables assigned in the score were significantly different between both groups (Table 1). In addition, a lower BMI (*p* = 0.027), low albumin levels (*p* < 0.001), and circumferential lesions (*p* < 0.001) were associated with high-risk patients. There was no difference between the groups regarding tumor location, cT, cN, Lauren’s type, or metastasis in other sites, besides the peritoneum.

Surgical, oncological, and postoperative outcomes of the patients according to risk group are presented in Table 2. The groups were different according to the surgical approach adopted (*p* = 0.001), and gastrectomy was more frequently performed in patients in the low-risk group (*p* = 0.036).

The occurrence of major POCs was higher in the high-risk group (*p* = 0.002). The rate of patients who received palliative chemotherapy was higher in the low-risk group (*p* < 0.001), as well as those who received the second and third line of treatment. There was no difference in terms of palliative radiotherapy between the two groups (*p* = 0.700).

In the high-risk group, 77.3% of patients died in <6 mo (*p* < 0.001). Score sensitivity, specificity, and positive and negative predictive values were 50%, 82%, 77%, and 58%, respectively.

### 3.2. Survival Analysis: Risk Groups and Subgroup Analysis According to Treatment

**Figure 2:**
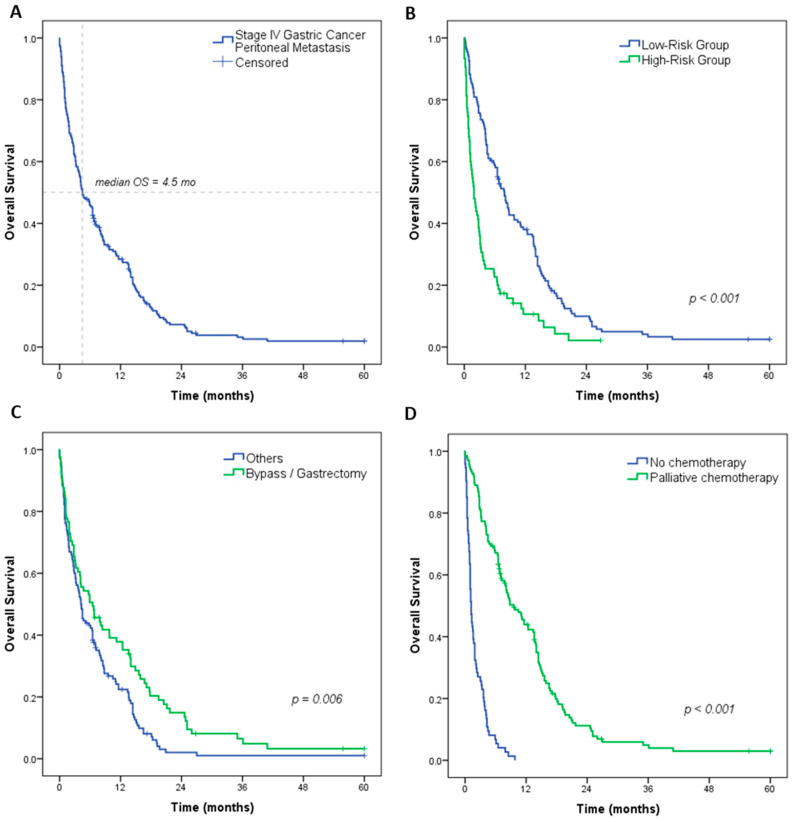
Overall survival (OS) of patients with stage IV gastric cancer with peritoneal carcinomatosis—(**A**) all patients; (**B**) OS according to risk groups for 6-month mortality; (**C**) surgical treatment; and (**D**) received palliative CMT.

The median survival for all patients was 4.5 months, and the estimated 5-year OS was 1.9%. The median OS was 7.9 and 1.9 months for low- and high-risk patients, respectively (*p* < 0.001) (Figure 2). According to the surgical treatment, patients undergoing palliative surgery (gastrectomy or bypass) had better survival compared with the other patients (6.5 vs. 4.2 mo, *p* = 0.006) (Figure 2). According to each of the approaches individually, the longest survival was achieved with gastrectomy, with a median OS of 12.4 months, and worse survival was observed in patients who underwent cytoreduction and jejunostomy (median OS of 2.4 mo and 2.3 mo, respectively). In addition, patients who received palliative CMT had significantly better survival compared with those who did not receive any line of CMT (median OS of 9.6 vs. 1.2 months, *p* < 0.001).

**Figure 3:**
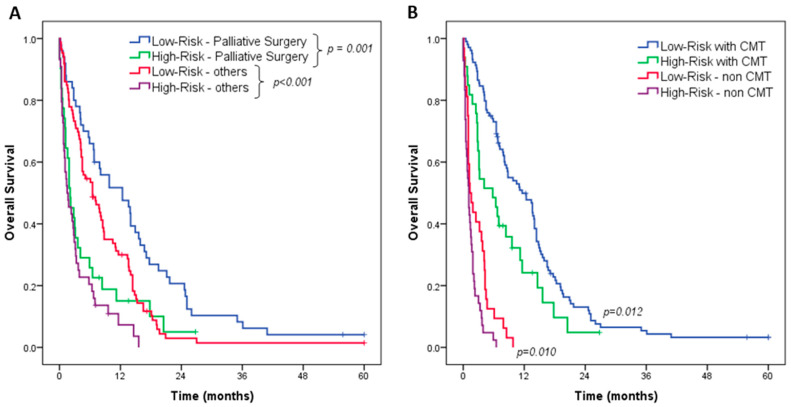
Overall survival (OS) of patients with stage IV gastric cancer with peritoneal carcinomatosis—subgroup analysis of risk groups according to treatment: (**A**) palliative surgery (gastrectomy or bypass) and (**B**) palliative CMT.

In the subgroup analysis, considering the risk groups in relation to the treatment performed, survival remained significantly different when comparing both the low- and high-risk groups. Among cases that underwent palliative surgery (gastrectomy or bypass), survival was significantly better among those classified in the low-risk group than in the high-risk group (median OS of 12.4 vs. 2.1 mo, *p* = 0.001). The median OS for the other procedures (diagnosis, jejunostomy, and cytoreduction) in the low- and high-risk groups was 6.5 and 1.5 months, respectively (*p* < 0.001). Among patients who underwent palliative CMT, the median OS survival in low-risk cases was better compared with high-risk cases (11.6 vs. 5.8 mo, *p* = 0.012). The same was observed among patients who did not receive chemotherapy, where OS was worse in high-risk than in low-risk cases (median OS of 1.3 vs. 1.1 mo, *p* = 0.010) (Figure 3).

## 4. Discussion

Peritoneal metastasis is common in GC, and its presence is still difficult to treat and carries a poor prognosis. Thus, the aim of the present study was to evaluate the survival outcomes of patients with clinical stage IV GC with peritoneal carcinomatosis and create a scoring system based on clinical variables to identify high-risk patients with poor physical status and extremely limited survival. Accordingly, the results of our study demonstrated that the risk scoring system for patients with PM that comprises clinical and tumor parameters had an ability to predict 6-month mortality as a short-term outcome. It may be useful in the individualization and selection of the most appropriate treatment, as some GCs with PC could benefit from more aggressive treatments based on expected survival. Furthermore, the prognostic significance of the risk group remained even in groups that underwent the same treatment, including palliative surgery and CMT, which can also help to predict prognosis after the therapeutic approach.

Unfortunately, the proportion of stage IV GC patients with peritoneal dissemination is not negligible. Among our metastatic adenocarcinoma, more than half of the patients had PC at the time of diagnosis (64%), which is similar to other cohorts, where peritoneal disease represents 58.9% of the cases [23]. Furthermore, 36.8% of cases have more than one metastatic site besides the peritoneum [23]. Herein, only 13.7% of cases had metastasis to a site other than the peritoneum.

Palliative CMT is still the mainstay of treatment for metastatic gastric adenocarcinoma, including in PM. But even with the improvement in the development of new chemotherapy regimens for stage IV GC, survival remains unsatisfactory. The survival in patients with gastric PM ranged from 2 to 9 months [24]. Similarly, in our study, the median OS was 4.5 mo for the entire cohort and 9.6 mo in patients who received palliative CMT, compared with only 1.2 mo for patients without CMT.

According to the palliative procedures, gastric bypass and jejunojejunostomy are also performed when there is disseminated PM in cases of unresectable GC [25,26]. In some patients, surgically treatable factors such as CG with outlet obstruction lead to intolerance of further systemic CMT [26]. Therefore, targeted surgical intervention can be used to palliate symptoms and facilitate the continuation of systemic therapy with acceptable postoperative outcomes. In this study, the rate of major POCs was 11.8% and 17.6% excluding cases that only underwent diagnostic laparoscopy. Studies reporting on quality of life and palliation indicate a possible benefit of such palliative gastrectomy [27]. Here, 13.3% of cases underwent gastrectomy. Although we do not have available data on quality of life, patients who underwent gastrectomy had significantly better survival compared with those who were not resected (12.5 mo), which was similar to that reported with HIPEC plus gastrectomy in a phase III clinical study (median of 11 mo) [9].

In the last few years, conversion therapy has been a subject of great interest. It is defined as a surgical treatment aiming for an R0 resection after CMT for tumors that are originally unresectable or marginally resectable for technical and/or oncological reasons. [13,15]. Today, indications for conversion therapy may include category 2 patients (absence of PM, marginally resectable metastases, or patients for whom surgery would not necessarily be the best choice), some category 3 patients (potentially unresectable PM, detectable macroscopical), and a small number of category 4 patients (non-curable metastases, with metastases to the peritoneum and other organs) [22].

Today, through the intraperitoneal administration of drugs, it is possible to reach the sites of PM, which was previously not possible with systemic CMT alone because of the peritoneal blood barrier [28,29]. This modality has resulted in improved survival, as well as an increase in conversion rates, but only in well-selected patients with GC with PM [4,5,11,12,17,28]. In a retrospective cohort study of patients with ECOG0/1 who had GC and PM with a median PCI of 4 (IQR, 2–9), one-third of the patients treated with laparoscopic HIPEC ultimately proceeded to cytoreduction and gastrectomy [12].

Certainly, strict patient selection is of utmost importance to ensure maximum benefit from these comprehensive treatment options, and one of the main aspects considered in the selection for intraperitoneal CMT and conversion surgery is the disease burden. For patients with PM from GC, a peritoneal cancer index (PCI) of a maximum of 10 to 12 has been suggested [4,5]. In our ongoing phase II clinical trial, a PCI of 12 was adopted as an inclusion criterion for the indication of peritoneal CMT followed by gastrectomy in patients with GC [14]. Unfortunately, in this retrospective study, we did not have a PCI available for all patients, so we were not able to verify whether the burden of peritoneal disease influenced early mortality and survival.

Besides the category that applies to stage IV GC and PCI, it is known that other aspects also serve as prognostic factors that are important to consider when choosing multimodal therapies for PM in an attempt to improve long-term survival [4]. Zheng et al. identified some potential subsets of patients with incurable GC who would benefit from palliative gastrectomy. Among the investigated variables, male sex, age of 50–59 years, Borrmann type III/IV, and peritoneal metastasis with D2 lymphadenectomy were identified as the factors that improve OS after gastrectomy [30]. In our study, the risk score was developed with nine pre-treatment variables related to patient baseline characteristics (age, sex, comorbidity), clinical/physical status (ASA, hemoglobin, NLR), and tumor characteristics (ascites, stenosis, size). The choice of these variables was based on characteristics presented as having a prognostic impact on patients with stage IV GC (age, sex, ASA, comorbidities) [3,30], in addition to variables that were identified in the univariate analysis as being significantly associated with mortality in the present cohort, such as hemoglobin levels, NLR, ascites, stenosis, and tumor size. Interestingly, the NLR and the presence of ascites on computed tomography (CT) were those with the highest scores (three points each).

The role of inflammation in cancer development is a well-known phenomenon, where the ratio of neutrophils and lymphocytes reflects the balance between pro- and anti-tumor immune activities [31]. A high NLR has already been reported as a factor associated with reduced survival in GC, including in IV patients [31].

Regarding the presence of ascites, the prognosis of GC with PM can be predicted through the degree of peritoneal dissemination of the disease. Honda et al. developed a pre-treatment CT ascites classification system as follows: Grade 0: absence of ascites in all sections; Grade 1: ascites only in the upper or lower abdominal cavity; Grade 2: ascites detected in the upper and lower abdominal cavities; and Grade 3 ascites extending continuously from the pelvic cavity to the upper abdominal cavity. This classification was able to predict the prognosis of patients with PC, where the average overall survival times were 16, 8.7, 5.4, and 3 months for ascites on CT grades 0, 1, 2, and 3, respectively [32]. In addition to ascites, we also included other characteristics that can reflect more advanced diseases, such as stenosis and tumor size.

Among the other variables included in the score, advanced age, ASA classification, presence of comorbidities, and low serum hemoglobin level are known to be related to a worse prognosis in patients with GC, especially because of lower adherence to chemotherapy [33]. Many patients with PC suffer from an interruption of CMT because of unplanned hospitalizations [23], and cases with poor physical status tend to develop complications during the treatment courses, which cause early death. Conversely, in those with good physical conditions, more active treatment can still bring a better prognosis. In a study that included 147 GC patients with PM, multivariate analysis demonstrated that a poor Eastern Cooperative Oncology Group (ECOG) performance score and severe PM were significantly associated with poor survival, while palliative CMT was an independent factor for favorable prognostic factors [34].

In our study, as expected, those GCs classified as high-risk were also associated with poor clinical status and the occurrence of POCs. Therefore, as the life expectancy of such patients is very short, aggressive surgical treatments are considered unreasonable, owing to the relatively high risk of postoperative morbidities that may delay CMT. This was also evidenced in our study, where the occurrence of POCs was associated with lower adherence to palliative CMT. Among our patients who had POCs, only 24% received CMT, compared with 70% among those without POCs.

In our cohort, the risk groups were also different in terms of some clinical characteristics, such as BMI and albumin levels, indicating a clinically impaired patient who might present a worse prognosis. Considering pathological characteristics, we expected that the high-risk group would have a predominance of GC with diffuse Lauren type and poorly differentiated histology, suggesting that a more aggressive disease could impair the prognosis or adherence to palliative treatments. However, similar to Chen et al., we did not find an association between Lauren type and survival [34]. Remarkably, the question of whether histological type can influence the indication for palliative systemic CMT or intraperitoneal CMT followed by surgery needs to be addressed. Although we found no differences between the risk groups, in the signet ring subtype GC with PM, the prognosis appears to remain poor irrespective of HIPEC and CRS [35]. Some authors reported that CG with the signet ring subtype showed an independent negative influence on survival [36].

Overall, we proposed a simple risk stratification into two groups based on a risk score, which was associated with mortality and, consequently, may serve as an additional parameter to predict patient outcomes. Noteworthily, the risk groups also served to stratify patients with different prognoses, even among those who underwent the same type of treatment. In fact, patients who underwent palliative surgery (gastrectomy and bypass) and those who received CMT had better survival in our series. Thus, we also evaluated the performance of the risk groups along these approaches. And, in both, the survival of the high-risk group was significantly worse compared with the low-risk group, showing the performance of the risk score stratification independent of the treatment applied.

The current study has some limitations. First, this is a retrospective study that only included patients in clinical stage IV with PM who underwent some surgical procedures. Therefore, possible confounding factors and selection bias are inevitable. Also, we include patients undergoing different types of treatment, which would be a possible bias. However, we believe that this limitation did not affect the results since the difference between the low- and high-risk groups was maintained in patients who underwent palliative surgery and received CMT—both being associated with decreased overall mortality [4,23,26].

Despite the aforementioned limitations, we performed a study in a well-characterized cohort of Western GC with PM to assess the prognostic factor in a group of patients generally overlooked by studies. All patients were treated in a single reference center in a real-world setting. Furthermore, all variables included in the score can be easily obtained through routine exams performed on patients with GC, which facilitates the applicability and implementation of the score.

Finally, the treatment of PM from GC continues to evolve, and understanding the factors that lead to worse outcomes in these patients is still required to guide the selection of the most appropriate treatment in an attempt to better control the disease. Since there is continuous progress in the field of CMT in stage IV GC, we intend to test the performance of our risk score in our patients included in an ongoing clinical trial with intraperitoneal CMT for GC (NCT05541146) [14]. In any case, the exact value and purpose of any scoring system need to be defined. Therefore, further independent assessments will be required to reach a validation and to recommend the prognostic scoring system for use in clinical practice and trials.

## 5. Conclusions

The scoring system developed with nine variables related to patient performance status and clinical data was able to distinguish GC with PM with a high risk of 6-month mortality. This may help to identify the patients most likely to benefit from more aggressive treatments and new therapeutic modalities. The score also served to stratify patients into different prognoses, even after treatment. Thus, it is a simple and reproducible score that may serve as a practical tool to identify the early death for stage IV GC with PC and help provide a more individualized treatment strategy. Accordingly, verifying and validating our findings in a large cohort of patients is necessary to confirm and guarantee the external validation of the results

## Figures and Tables

**Table 1:** Clinical characteristics and staging of patients with stage IV gastric cancer with peritoneal carcinomatosis, according to risk groups for 6-month mortality.

Variable		Low Risk	High Risk	*p* *
		n = 136 (%) ^#^	n = 75 (%) ^#^	
**Sex**				0.043
	Female	46 (33.8)	36 (48)	
	Male	90 (66.2)	39 (52)	
**Age (years)**				<0.001
	Mean (SD)	58 (12.2)	65 (12.6)	
**Body mass index (Kg/m^2^)**				0.027
	Mean (SD)	22.8 (4.7)	21.4 (4.0)	
**ASA classification**				0.002
	I/II	93 (68.4)	35 (46.7)	
	III/IV	43 (31.6)	40 (53.3)	
**Charlson comorbidity index (CCI)**				0.006
	CCI 0	116 (85.3)	52 (69.3)	
	CCI > I	20 (14.7)	23 (30.7)	
**Hemoglobin (g/dL)**				<0.001
	Mean (SD)	11.5 (2.1)	9.9 (2.1)	
**Albumina (g/dL)**				0.002
	Mean (SD)	3.7 (0.6)	3.4 (0.8)	
**Neutrophil** **–** **lymphocyte ratio**				<0.001
	Mean (SD)	3.04 (3.36)	8.00 (7.07)	
**Tumor location**				0.604
	Lower	53 (39)	35 (46.7)	
	Middle	50 (36.8)	21 (28)	
	Upper	20 (14.7)	11 (14.7)	
	Plastic linite	13 (9.6)	8 (10.7)	
**Tumor size (cm)**				0.010
	Mean (SD)	7.7 (3.7)	9.1 (3.9)	
**Histological type**				0.834
	Intestinal	20 (14.7)	11 (14.7)	
	Diffuse/mixed	58 (42.6)	29 (38.7)	
	Adenocarcinoma na	58 (42.6)	35 (46.7)	
**Differentiation grade**				0.365
	G1/G2	15 (11)	12 (16)	
	G3	63 (46.3)	28 (37.3)	
	Adenocarcinoma (not specified)	58 (42.6)	35 (46.7)	
**cT**				0.153
	cT3	3 (2.2)	2 (2.7)	
	cT4a	76 (55.9)	33 (44)	
	cT4b	54 (39.7)	40 (53.3)	
**cN**				0.153 *
	cN0	2 (1.5)	0 (0)	
	cN1	6 (4.4)	1 (1.3)	
	cN2	19 (14)	5 (6.7)	
	cN3	109 (80.1)	69 (92)	
**cM**				0.480
	Only peritoneal	119 (87.5)	63 (84)	
	Peritoneum and other sites	17 (12.5)	12 (16)	
**Stenosis**				<0.001
	No	87 (64)	22 (29.3)	
	Yes	49 (36)	53 (70.7)	
**Ascites on CT Scan**				<0.001
	No	93 (68.4)	29 (38.7)	
	Yes	43 (31.6)	46 (61.3)	
**Circumferential tumor**				<0.001
	No	56 (41.2)	10 (13.3)	
	Yes	80 (58.8)	65 (86.7)	

SD, standard deviation; CT, computed tomography. ^#^ The percentages expressed in the table refer to the total “n” of each column; * chi-square was used for categorical variables and the *t*-test for continuous variables.

**Table 2:** Treatment characteristics and outcomes of patients with stage IV gastric cancer with peritoneal carcinomatosis, according to risk groups.

Variable		Low Risk	High Risk	*p* *
		n = 136 (%) ^#^	n = 75 (%) ^#^	
**Type of surgery**				0.001
	Diagnostic laparoscopy	54 (39.7)	15 (20)	
	Bypass	27 (19.9)	26 (34.7)	
	Gastrectomy (total/subtotal)	23 (16.9)	5 (6.7)	
	Jejunostomy	28 (20.6)	27 (36)	
	Cytoreduction	4 (2.9)	2 (2.7)	
**Gastrectomy**				0.036
	No	113 (83.1)	70 (93.3)	
	Yes	23 (16.9)	5 (6.7)	
**Postoperative complications (POCs)—All**				0.002
	non-POC/Clavien I-II	127 (93.4)	59 (78.8)	
	Clavien III–V	9 (6.6)	16 (21.3)	
**Postoperative complications (POCs)—excluding diagnostic laparoscopy**				0.008
	non-POC/Clavien I-II	74 (90.2)	44 (73.3)	
	Clavien III–V	8 (9.8)	16 (26.7)	
**Hospitalization time (days)**				0.586
	Mean (SD)	5.9 (7.4)	6.4 (5.3)	
**Palliative chemotherapy**				<0.001
	No	32 (23.5)	42 (56)	
	Yes	104 (76.5)	33 (44)	
**Chemotherapy—lines**				
	1st line	104 (76.5)	33 (44)	<0.001
	2nd line	52 (38.2)	9 (12)	<0.001
	3rd line	15 (11)	1 (1.3)	0.012
**Death < 6 months**				<0.001
	No	79 (58.1)	17 (22.7)	
	Yes	57 (41.9)	58 (77.3)	

SD, standard deviation; ns, not specified. ^#^ The percentages expressed in the table refer to the total “n” of each column; * chi-square was used for categorical variables, and the *t*-test for continuous variables.

## Data Availability

No additional data are available.

## Supplementary Materials

The following supporting information can be downloaded at https://www.mdpi.com/article/10.3390/medsci12020030/s1, Table S1: Data of patients with stage IV gastric cancer with peritoneal carcinomatosis—all patients; Table S2: Binary logistic regression of risk factors related to death within 6 months—variables included in the score; Table S3: Variables and points used in the risk score calculation.
