# Supplementary material for: Risk Scoring System to Predict Mortality in Gastric Cancer with Peritoneal Carcinomatosis
Source: Med Sci (Basel). 2024 Jun 9;12(2):30. doi: 10.3390/medsci12020030 (PMC11205632; doi:10.3390/medsci12020030)
Supplement: Supplementary file 1 [file medsci-12-00030-s001.zip › medsci-2996000-supplementary.pdf]

medical sciences

MDPI

Original Research

Risk Scoring System to Predict Mortality in Gastric Cancer with Peritoneal Carcinomatosis

Marina Alessandra Pereira [1], Marcus Fernando Kodama Pertille Ramos [1], Amir Zeide Charruf [1], André Roncon Dias [1] and Ulysses Ribeiro, Jr. [1]

Supplementary Materials: The following supporting information can be downloaded at: www.mdpi.com/xxx/s1, **Table S1**: Data of patients with stage IV gastric cancer with peritoneal carcinomatosis – all patients; **Table S2**: Binary logistic regression of risk factors related to death within 6 months – variables included in the score; **Table S3**: Variables and points to risk score calculation.

Table S1. Data of patients with stage IV gastric cancer with peritoneal carcinomatosis – all patients.

Variables	n = 211	%
**Sex**		
Female	82	38.9
Male	129	61.1
**Age (years)**		
Mean (SD)	60.5 (12.8)	
**Body Mass Index (Kg/m²)**		
Mean (SD)	22.3 (4.5)	
**ASA Classification**		
I / II	128	60.7
III / IV	83	39.3
**Charlson Comorbidity Index (CCI)**		
CCI 0	168	79.6
CCI > I	43	20.4
**Hemoglobin (g/dL)**		
Mean (SD)	10.9 (2.2)	
**Albumin (g/dL)**		
Mean (SD)	3.6 (0.7)	
**Neutrophil-Lymphocyte Ratio**		
Mean (SD)	4.84 (5.56)	
**Tumor Location**		
Lower third	88	41.7
Middle third	71	33.6
Upper third	31	14.7
Plastic linite	21	10.0
**Tumor size (cm)**		
Mean (SD)	8.2 (3.8)	
**Histological Type**		

Intestinal	31	14.7
Diffuse / mixed	87	41.2
Adenocarcinoma (not specified)	93	44.1
**Differentiation Grade**		
G1/G2	27	12.8
G3	91	43.1
Adenocarcinoma (not specified)	93	44.1
**cT**		
cT3	8	3.8
cT4a	109	51.7
cT4b	94	44.5
**cN**		
cN0	2	0.9
cN1	7	3.3
cN2	24	11.4
cN3	178	84.4
**cM1**		
Only peritoneal	182	86.3
Peritoneum and other sites	29	13.7
**Stenosis**		
No	109	51.7
Yes	102	48.3
**Ascites on CT Scan**		
No	122	57.8
Yes	89	42.2
**Circumferential Tumor**		
No	66	31.3
Yes	145	68.7
**Type of Surgery**		
Diagnostic laparoscopy	69	32.7
Bypass	53	25.1
Gastrectomy (total/subtotal)	28	13.3
Jejunostomy	55	26.1
Cytoreduction	6	2.8
**Postoperative Complications (POC)**		
non-POC/Clavien I-II	186	88.2
Clavien III - V	25	11.8
**Hospitalization time (days)**		
Median (IQR)	4 (2 - 8)	
**Palliative chemotherapy**		
No	74	35.1
Yes	137	64.9
**Death < 6 months**		

No	96	45.5
Yes	115	54.5
**Risk Score**		
Low-Risk (<9)	136	64.5
High-Risk (≥9)	75	35.5

SD, standard deviation; IQR, interquartile range

Table S2. Binary logistic regression of risk factors related to death within 6 months – variables included in the score.

**Variables**	**Exp(B)**	**95% CI**
Age ≥60 (vs <65 years)	1.63	0.83 - 3.18
Female (vs male)	1.90	0.98 - 3.68
Neutrophil-Lymphocyte Ratio (NLR) ≥3.85 (vs NLR <3.85)	2.73	1.37 - 5.46
Hemoglobin (Hb) <11 g/dL (vs Hb ≥ 11g/dL)	1.43	0.75 - 2.72
Charlson Comorbidity Index (CCI) ≥1 (vs CCI 0)	0.97	0.40 - 2.34
ASA III/IV (vs ASA I/II)	1.34	0.67 - 2.65
Stenosis (vs absent)	1.42	0.75 - 2.67
Ascites on Computed Tomography (CT) Scan (vs absent)	3.34	1.73 - 6.46
Tumor size ≥8 cm (vs < 8cm)	1.13	0.60 - 2.13

Table S3. Variables and points to risk score calculation.

**Variable**	**Category**	**Points**
Neutrophil-Lymphocyte Ratio (NLR)	≥3.85	3
Ascites on Computed Tomography (CT) Scan	Present	3
Hemoglobin	<11 g/dL	2
Age	≥60 years	2
Sex	Female	2
Stenosis	Present	2
Tumor Size	≥8 cm	1
ASA	III/IV	1
Charlson Comorbidity Index (CCI)	CCI ≥1	1
**Total**		**17**
